# Formal education, previous interaction and perception influence the attitudes of people toward the conservation of snakes in a large urban center of northeastern Brazil

**DOI:** 10.1186/s13002-016-0096-9

**Published:** 2016-06-20

**Authors:** Luan Tavares Pinheiro, João Fabrício Mota Rodrigues, Diva Maria Borges-Nojosa

**Affiliations:** Núcleo Regional de Ofiologia da Universidade Federal do Ceará (NUROF-UFC), Depto. Biologia, Universidade Federal do Ceará, Campus do Pici, Fortaleza, CE 60440-554 Brazil; Programa de Pós-Graduação em Biologia de Vertebrados, Pontifícia Universidade Católica de Minas Gerais, Belo Horizonte, MG 30535-610 Brazil; Programa de Pós-Graduação em Ecologia e Evolução, Departamento de Ecologia, Instituto de Biociências da Universidade Federal de Goiás, Goiânia, GO 74001-970 Brazil

**Keywords:** Ethnoherpetology, Ethnozoology, Fear, Gender, Environmental Education, Animal Conservation, Fortaleza, Ceará, Reptiles, Squamata

## Abstract

**Background:**

The attitudes and perceptions of people toward animals are influenced by sociodemographic factors, such as formal education and gender, and by personal experience. Understanding these interactions is critical for the establishment of conservation strategies for animals that have conflictual relationships with humans, such as snakes. Our study aims to explain how perceptions and the human fear of snakes vary and are influenced by formal education and gender. In addition, it aims to show how prior interaction with these animals influence these perceptions and the human fear toward snakes and how these perceptions and fear influence the importance of conservation of these animals.

**Methods:**

We collected data from June 2010 to December 2013 using questionnaires given to 1142 visitors of a scientific serpentarium (Núcleo Regional de Ofiologia da Universidade Federal do Ceará) in the municipality of Fortaleza, northeastern Brazil.

**Results and Discussion:**

Negative perceptions toward snakes were less frequent according to an increase in levels of schooling. Women had more negative perceptions and were more afraid of snakes than were men. Prior interaction with snakes decreased the occurrence of negative perceptions and reduced the level of human fear of these animals. People with negative perceptions classified the conservation of snakes as not important and were more afraid of these animals. Understanding the relationship between sociodemographic factors, prior experiences, perceptions, fear, and the importance given to conservation can help to better understand human attitudes toward snakes.

**Conclusions:**

Environmental education activities considering gender differences, involving preliminary interaction with snakes and focusing on priority targets identified in our study, such as people with low formal education, can increase the efficiency of measures for the conservation of these animals.

**Electronic supplementary material:**

The online version of this article (doi:10.1186/s13002-016-0096-9) contains supplementary material, which is available to authorized users.

## Background

The records of interactions between animals and people are very old, as evidenced by numerous indications, such as cave paintings, that report various types of human–animal interactions [1]. However, this relationship was not always harmonious, with conflicts occurring when animals are considered competitors or dangerous, a fact that may reflect a situation where the animal really endangers human lives or due cultural aspects, such as beliefs and religious traditions [2]. In addition, human attitudes toward animals are strongly influenced by perceptions and personal experience related to them [3–5]. There are many factors that can affect the perceptions and attitudes of people toward animals. These factors act together and include specific animal attributes (shape, size, behaviour, use by humans), individual human attributes (gender, age, formal education, urban or rural residence) and cultural influences (religious, myths) [4]. Fear related to animals is another important factor that can influence the attitudes of people towards animals. The fear of certain species can be explained by evolutionary approaches [6], cultural influences [4, 7] and social learning [8].

Previous experience with animals can also influence perceptions and actions toward them. This may be seen in surveys of visitors to museums and zoos, which report that the attitudes of people toward the animals changed after visiting these places [9, 10]. Besides, some studies also report that positive interactions with animals help in the treatment of phobias related mainly to snakes [11–13].

Ethnozoology seeks to understand these relations between humans and animals [14]. Many studies try to explain how these attitudes and perceptions toward animals are influenced by sociodemographic factors, such as formal education and gender. Some studies suggest that formal education can have an effect on people’s attitudes toward animals, indicating that a higher levels of schooling of the person is reflected in more positive attitudes held by the person [15, 16]. Ceríaco [17] suggested that people with higher levels of schooling have fewer misperceptions about the herpetofauna linked to folklore, which could be explained by their greater formal knowledge when compared to people with low levels of schooling. Among the sociodemographic factors, gender presents a clearer pattern in relation to perceptions: women have more negative attitudes and fear toward many animal groups, such as bats and spiders [18], carnivorous predators [19] and snakes [20].

Ethnoherpetology, a subdivision of ethnozoology, focus in human interactions with reptiles and amphibians [21]. Ethnoherpetological studies in Brazil, address different approaches, such as traditional medicinal use [22, 23], folklore [15, 24] and hunting [25, 26]. Despite their different aims and approaches, these studies are mainly descriptive, showing list of hunted species, popular beliefs or lists of reptiles used in traditional folk medicine [14]. In this sense, there are few studies in Brazil that address the conflicting relationship with these animals testing hypotheses for their possible explanations (but see [27]).

Snakes have historically been persecuted and the target of negative attitudes in many countries [15, 17, 20, 28]. In rural areas of Brazil, the most common attitude of people when they find snakes is simply to kill them [25, 29]. In a study conducted in northeastern Brazil, all 124 interviewed hunters said that they kill any snake they encounter in their daily lives [25]. This shows that the current situation found in Brazil involves indiscriminate killing of several species of snakes, both venomous and non-venomous. The causes of these negative attitudes are diverse: religious factors, such as biblical quotations that picture the snake as “villains”, cultural issues such as myths involving these animals [15], evolutionary influences, evidenced by the high prevalence of snake fear in experiments involving humans and monkeys [6, 30], and personal experience with snakes [8]. Therefore, the interaction between humans and snakes must be taken into account for the development and adoption of effective conservation strategies for these animals [2].

Thus, our study aims to understand how perceptions and fear of humans toward snakes vary according to formal education and gender, how previous interactions with these animals influence these perceptions and fear, and finally, how the perceptions and fear influence the importance of the conservation of snakes. With this, we can provide a basis for the development of future conservation actions for these animals, focusing on specific human groups with the greatest conflicts with snakes, acting directly on the roots of the existence of a negative relationship.

## Methods

### Data collection

The study group was composed of visitors from extension projects of the Núcleo Regional de Ofiologia da Universidade Federal do Ceará (NUROF-UFC), a scientific serpentarium legalised by the Instituto Brasileiro do Meio Ambiente e dos Recursos Naturais Renováveis (IBAMA) and the Instituto Chico Mendes de Conservação da Biodiversidade (ICMBio), located at the Campus do Pici, Fortaleza, Ceará, northeastern Brazil. Data were collected between July 2010 and December 2013 using a structured interview with a questionnaire (Additional file 1) [31]. Visitation in NUROF-UFC was composed by organized groups of students (primary, secondary and higher education), police, and health professionals, among others. The visitants were driven to NUROF-UFC by their educational institutions or work company and the visitation to NUROF-UFC was part of the curricular program of these visitant groups.

In NUROF-UFC activities, there is a didactic exhibition open to public visitation where living snakes, specimens preserved in alcohol and various sources of information about these vertebrates may be found. In the visitation routine for our study, the group of visitors was initially led to the didactic exhibition of the NUROF-UFC. During this first contact, the students responsible for guiding the visit did not interact with the visitors through information or answering questions. After visitors had this first visual contact with snakes, they were led to a classroom where we presented a questionnaire to them (Additional file 1) with questions about their perceptions toward snakes. Each visitor replied to the questionnaire only once.

In the questionnaire, one of the questions was about the perception of the visitors when they saw snakes in NUROF-UFC (Question 2 of Additional file 1). In the question about the perception of visitors, we used the word “feeling” because it is more understandable to the general public. The other question was about the intensity of the fear that visitors felt about the snakes, where the items were “no”, “little”, “very” or “panic”(Question 8 of Additional file 1). We dealt with fear separately due to the great influence of this feeling on people’s attitude toward animals. Other questions were related to prior interaction with snakes before visiting the NUROF-UFC (Question 5 of Additional file 1). We considered prior interaction to be any visual contact with snakes in the natural environment or in exhibitions and zoos. Visitors were also asked whether they considered the conservation of snakes as important or not (Question 10 of Additional file 1). We used the word “preservation” in this question to be more understandable to the general public, but throughout this paper we use the term “conservation”.

Participation in the survey was not obligatory, and prior to data collection, the visitors were informed about the study objectives and advised to not identify themselves in the questionnaires (names or document number). No time limit was given for completing the questionnaire. This research followed ethical precepts and was formally registered in the Pro Extension Rectory of the Federal University of Ceará (Protocol – CH00.2005.PG.0009).

### Data Analysis

For all analyses we considered only residents of the metropolitan region of Fortaleza, Ceará, Brazil. We categorised the levels of schooling of participants on a gradient scale according to the time of study, following the Ministry of Education of Brazil’s guidelines. The first grade of elementary school was equivalent to one year of study, 5th grade, five years and so on until the 9th grade. We adopted the same criterion for the high school, where the first year amounted to 10 years of study, the second, 11 years, and the third, 12 years. We assigned a study time of 15 years for higher education (graduation), for master’s degree, 17 years, and for a PhD, 20 years.

The perceptions described when viewing snakes in NUROF-UFC were independently categorised as “positive”, “negative” and “unrated” (those considered “meaningless” or those that had no positive or negative aspects) by two researchers. Then, the two classifications were compared, and the perceptions classified differently in both classifications were allocated into a single category with an agreement between the two researchers. We did not use perceptions categorised as “unrated” in the statistical analyses explained below.

Due to the difficulty distinguishing between fear levels “no” and “little” and between “very” and “panic”, we grouped the responses regarding the level of fear related to snakes into two groups: the “no/little” group, and the “very/panic”. Due to a change in the questionnaire in the beginning of data collection, we disregarded 147 responses regarding this question in the analysis.

We used a logistic regression to test the influence of levels of schooling on negative perceptions and fear. We used the Chi-square test to assess the existence of dependence between: 1) negative perceptions and gender, 2) fear and gender, 3) negative perceptions and prior interaction, 4) fear and prior interaction, 5) negative perceptions and importance of conservation, 6) fear and importance of conservation.

We analysed the variables using a significance level of *P* < 0.05. The analyses were performed in the software R ver. 3.0.2 (Development Core Team, 2013).

## Results

**Fig. 1:**
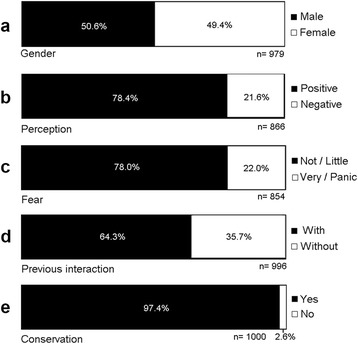
Responses of the questions answered by visitors of the NUROF-UFC (Núcleo Regional de Ofiologia da Universidade Federal do Ceará) during the period of 2010 to 2013 about **a** gender; **b** perception toward snakes; **c** fear toward snakes; **d** previous interaction with snakes; **e** importance to conserve snakes

We collected data of 1142 persons, but only the data of the residents of metropolitan region of Fortaleza, 1006 persons, were considered in this study. The age of the visitants was between 11 and 54 years (mean ± standard deviation 19.7 ± 7.2 years), and we had almost the same proportion of males and females among the respondents (Fig. 1-a). Majority of visitants had positive perceptions toward snakes (Fig. 1-b). The three perceptions most cited by the participants, in descending order of citation (number of citations in parentheses) for each category, were “curiosity” (223), “interest” (94), and “admiration” (54) for positive perceptions, and “fear” (114), “alarmed” (12) and “disgust” (7), for negative perceptions. “Normal”, “indifference” and “emotion” are examples of perceptions not categorised as positive or negative. Most people had no or few fear of snakes (Fig. 1-c), and the majority of respondents had prior interaction with these animals (Fig. 1-d). About the importance of snake conservation, the vast majority reported that conserving snakes is important (Fig. 1-e).

**Fig. 2:**
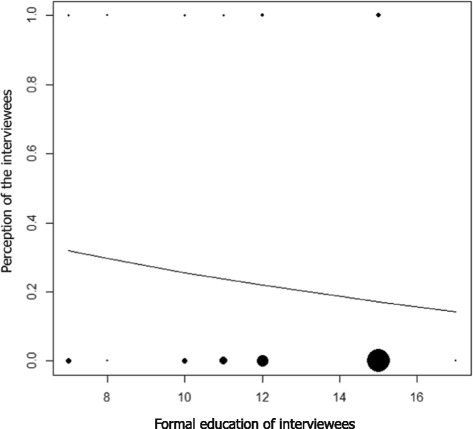
Relationship between formal education and perceptions of NUROF-UFC (Núcleo Regional de Ofiologia da Universidade Federal do Ceará) visitors toward snakes during the period between 2010 and 2013. A value of 1 was assigned to perceptions considered negative (fear, disgust, and alarmed, for example) and 0 to perceptions considered positive (curiosity, interest, and admiration, for example). The dashed line in the centre represents a trend based on the values expected by the logistic regression model. The size of the points is proportional to the sample size

**Fig. 3:**
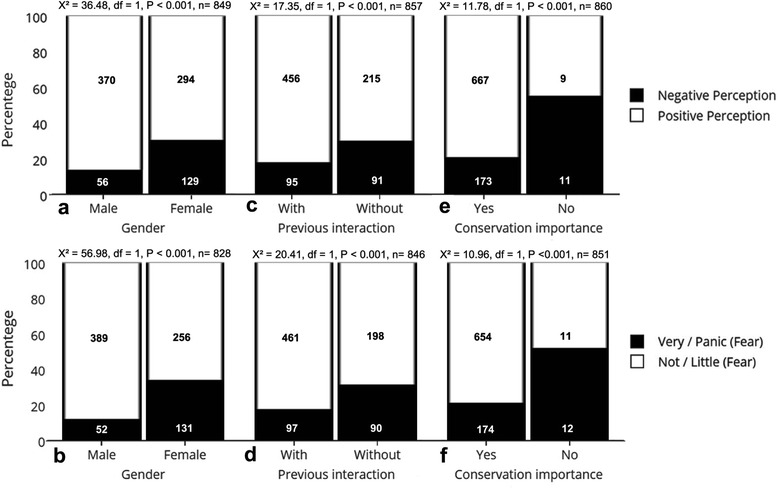
Relationship between **a** gender and perception, **b** gender and fear, **c** previous interaction and perception, **d** previous interaction and fear, **e** importance of snake conservation and perception, **f** importance of snake conservation and fear from the visitors of the NUROF-UFC (Núcleo Regional de Ofiologia da Universidade Federal do Ceará) during the period of 2010 to 2013

The frequency of negative perceptions decreased with increases in the level of schooling of visitors (z = -3.39, *P* < 0.001, *N* = 862) (Fig. 2). Fear related to snakes was not influenced by the level of schooling of people (z = 1.21; *P* = 0.2240; *N* = 850). Women had more negative perceptions (Fig. 3-a) and were more afraid of snakes than men (Fig. 3-b). People who had no prior interaction with these animals had more negative perceptions (Fig. 3-c) and more fear (Fig. 3-d). The importance that people give to the conservation of snakes was negatively influenced by negative perceptions (Fig. 3-e) and fear (Fig. 3-f).

## Discussion

Many recent studies have addressed the attitudes and the relationship between humans and snakes in Brazil [15, 24, 27, 29, 32]. However, this is the first study to demonstrate, on the basis of a large sampling effort, that formal education influences the way people perceive and interact with these animals. We found that gender, previous interaction and the importance of conservation have a similar effect on the fear and perceptions, as evidenced in Fig. 3. This result could be expected given that fear and negative perceptions are probably related. We also presented how the prior interaction with snakes relates to perceptions, and how these perceptions relate to the importance that people give to the conservation of snakes. Finally, we also observed that the gender of people influences perceptions regarding these animals. As a result, conservation projects should consider these sociodemographic questions that strongly influence the human–snake relationship.

Overall, most visitors showed positive perceptions, little fear toward snakes and considered important the conservation of snakes. These positive results may have been partly influenced by certain factors. Interviewees were in a large urban area where there is a greater presence of media and access to information about conservation than in rural areas. Furthermore, in urban areas there is a lower school evasion than in rural areas in Brazil [33]. The importance of this difference may be proven by comparing studies addressing the relationship between humans and snakes in urban areas ([12], this study), and in rural areas [25, 27, 29] and in both environments simultaneously [17], making clear the greater presence of positive attitudes and perceptions in urban areas. However, we have also to consider that although the visitors went to the serpentarium as part of their curricular program, these people were willing to visit a place with snakes and learn more about these animals, which could also explain these positive results. The fact that the visitors were in a laboratory with the presence of biologists at the time of sampling could also have inhibited them to express more negative responses.

The negative relationship found in our study between negative perceptions toward snakes and the levels of schooling may be explained due to the increase in knowledge to be able to dispel some myths related to snakes. These myths are one of the factors that generate negative perceptions related to these animals [15, 17, 27]. In addition, a better understanding of the biology of snakes, their ecological importance and a general knowledge of snake bites, which can be acquired through formal education, may also explain why people with higher levels of schooling tend to have fewer negative perceptions of these animals [29]. Thus, investing in education may be an important solution to try to reduce the killing that has affected snakes over time, especially in rural areas, where there is a greater contact with these animals and where the level of formal education and access to information are generally lower than in urban areas. In this context, projects of environmental education, such as those developed by NUROF-UFC, are also very important to show the ecological relevance of snakes and elucidate myths that erroneously give potential risks to snakes [27, 32]. However, there was no influence of level of formal education on the level of fear, showing that this feeling may be probably more influenced by other factors, such as prior interaction, as showed in our study (Fig. 3).

Regarding gender, women have more negative perceptions and more fear of snakes than do men, a similar pattern to that found with snakes [20] and with bats and spiders [18, 34]. One of the possible reasons for this gender difference is that women believe more in myths about snakes than do men [20] and men generally have more interest in animals considered less charismatic to Western culture, such as snakes, spiders and bats [35, 36] and wildlife [19]. Women tend to present more fear than men in relation to different situations (e.g., darkness, enclosed) and animals [37, 38]. This general gender difference has received many possible explanations, as hormonal influences, genetic factors, sociocultural influences among several others [39]. Regarding snakes, gender difference in fear appears in childhood and can be maintained until the adulthood, and a possible sexual division of labor in a hunter-gatherer approach may explain the origin of this sexual difference [40]. However some studies criticize the stereotyped view of the sexual division of labor and indicate that the role of women in hunting may have changed according to the different geographical environments where the ancient societies lived [41, 42]. Therefore the reasons for the gender differences in fear of snakes remain inconclusive and more studies are needed to address this issue.

The interaction with animals, by physical contact with real animals and models or through eye contact, is used to treat people with phobias to snakes, producing positive results, effectively reducing the fear of people toward these animals [11–13]. This effect is evident in our study, since we found a negative relationship between the previous interaction with these animals and negative perceptions and the level of fear. Contact with animals in museums and zoos also influences the interest and knowledge of people regarding the fauna [9, 10, 20, 43]. This was recently demonstrated by Moss et al. [44] in a survey of 5661 visitors from 26 zoos and aquariums in 19 countries around the globe, highlighting the importance of these sites in the maintenance and conservation of biodiversity. However, continued actions are necessary for these activities to be able to influence the attitudes of visitors [45]. Therefore, the recovery and better use of recreational areas that allow for contact with animals can play an important role in the conservation of local biodiversity.

The rapid expansion of most major urban centres and the consequent deforestation of natural areas for civil construction have led the population of those places to have dwindling contact with species of wild animals. As previously discussed, the low level of interaction between animals and the human population leads to a higher rate of negative perceptions and fear among the population. Rejected animals, such as snakes, suffer even more in this type of interaction [15, 17, 27, 28]. Thus, it is important to understand how people perceive and deal with these animals and whether they are conscious of the importance of conserving the snakes.

## Conclusions

Our study showed that formal education is important in changing the attitude of people in relation to snakes and that although most people consider the conservation of snakes to be important, this importance is influenced by the perceptions and fear toward these animals. Thus, understanding the relationship between sociodemographic factors, previous experiences, perceptions, fear, and the importance given to conservation can help to understand people's attitudes toward snakes. As a result, the adoption of environmental education activities considering gender differences, involving preliminary interaction with snakes and focusing on priority target identified in our study, such as people with low formal education, can increase the efficiency of actions that seek the conservation of snakes.

## Additional file

Additional file 1: Questionnaire used in the data collection NUROF-UFC visitors in the 2010–2013. (PDF 198 kb)
