# Supplementary material for: Formal education, previous interaction and perception influence the attitudes of people toward the conservation of snakes in a large urban center of northeastern Brazil
Source: J Ethnobiol Ethnomed. 2016 Jun 20;12:25. doi: 10.1186/s13002-016-0096-9 (PMC4915046; doi:10.1186/s13002-016-0096-9)
Supplement: Additional file 1: — Questionnaire used in the data collection NUROF-UFC visitors in the 2010–2013. (PDF 198 kb) [file 13002_2016_96_MOESM1_ESM.pdf]

Additional file 1. Questionnaire used in the data collection NUROF-UFC visitors in the 2010–2013.

QUESTIONNAIRE - Translate version (English)

01) Age: ________.
Sex: [ ] Male [ ] Female
Level of Education:
Basic Education: [ ]1ª [ ]2ª [ ]3ª [ ]4ª [ ]5ª [ ]6ª [ ]7ª [ ]8ª [ ]9ª
Secondary School: [ ] 1º [ ] 2º [ ] 3º
Higher Education: [ ] Graduation [ ] Masters Degree [ ] Doctorate

02) Write in a word your feeling when you see snakes in the exhibition of the NUROF:

______________________.

03) Have you ever heard of NUROF-UFC before this visit?
[ ] YES [ ] NO

04) Do you know the name of any snake?
[ ] YES / Which one?_______________________.
[ ] NO

05) Have you ever faced a snake before your visitation in NUROF-UFC?
[ ] YES / Where? ___________________. What snake did you see? _______________.
[ ] NO

06) Do you know what to do if you find a snake that is outside of their natural habitat, a house, for example?
[ ]YES / What to do? ____.
[ ]NO

07) Have you ever saw someone killing a snake?
[ ]YES [ ]NO

08) Are you afraid of snakes?
[ ]NO [ ] LITTLE [ ] VERY [ ] PANIC

09) What subject related to snakes raises more your curiosity?
[ ] REPRODUCTION [ ] FEEDING [ ] DIVERSITY
[ ] ACCIDENT RISK [ ] MYTHS AND LEGENDS

10) Do you think that the preservation of snakes is important?
[ ] YES [ ] NO

Thanks!

Additional file 1. Questionnaire used in the data collection NUROF-UFC visitors in the 2010–2013.

QUESTIONNAIRE – Orginal version (Brazilian Portuguese)

01) Idade: ________.
Sexo: [ ] Masculino [ ] Feminino
Escolaridade:
Ens. Fundamental: [ ]1ª [ ]2ª [ ]3ª [ ]4ª [ ]5ª [ ]6ª [ ]7ª [ ]8ª [ ]9ª
Ens. Médio: [ ] 1º [ ] 2º [ ] 3º
Ens. Superior: [ ] Graduação [ ] Mestrado [ ] Doutorado

02) Escreva em uma palavra seu sentimento ao ver as serpentes na exposição do NUROF:

______________________.

03) Você já tinha ouvido falar do NUROF antes dessa visita?
[ ] SIM [ ] NÃO

04) Você sabe o nome de alguma serpente?
[ ] SIM / Qual?_______________________.
[ ] NÃO

05) Você já tinha se deparado com uma serpente antes da visita ao NUROF?
[ ] SIM / Onde foi? ___________________. Qual serpente você viu? _______________.
[ ] NÃO

06) Você sabe o que fazer ao se deparar com uma serpente que esteja fora de seu habitat natural, uma casa, por exemplo?
[ ]SIM / O que fazer? ____.
[ ]NÃO

07) Você já presenciou alguém matando uma serpente?
[ ]SIM [ ]NÃO

08) Você tem medo de serpentes?
[ ]NÃO [ ] POUCO [ ] MUITO [ ] PÂNICO

09) O que relacionado às serpentes lhe desperta mais curiosidade?
[ ] REPRODUÇÃO [ ] ALIMENTAÇÃO [ ] DIVERSIDADE
[ ] RISCO DE ACIDENTES [ ] MITOS E LENDAS

10) Você acha que a preservação das serpentes é importante?
[ ] SIM [ ] NÃO

Obrigado!
